# Re-analysis of mobile mRNA datasets raises questions about the extent of long-distance mRNA communication

**DOI:** 10.1038/s41477-025-01979-x

**Published:** 2025-04-16

**Authors:** Pirita Paajanen, Melissa Tomkins, Franziska Hoerbst, Ruth Veevers, Michelle Heeney, Hannah Rae Thomas, Federico Apelt, Eleftheria Saplaoura, Saurabh Gupta, Margaret Frank, Dirk Walther, Christine Faulkner, Julia Kehr, Friedrich Kragler, Richard J. Morris

**Affiliations:** 1 Computational and Systems Biology, John Innes Centre, Norwich, UK; 2 School of Integrative Plant Science, Cornell University, Ithaca, NY USA; 3 Cell and Developmental Biology, John Innes Centre, Norwich, UK; 4 Department II, Max Planck Institute of Molecular Plant Physiology, Potsdam-Golm, Germany; 5 Department of Biology, Institute for Plant Sciences and Microbiology, University of Hamburg, Hamburg, Germany; 6 Present Address: Curtin Medical School, Curtin Health Innovation Research Institute (CHIRI), Curtin University, Perth, Western Australia Australia

**Keywords:** Plant signalling, Bioinformatics, Sequencing, Plant physiology

## Abstract

Short-read RNA-seq studies of grafted plants have led to the proposal that thousands of messenger RNAs (mRNAs) move over long distances between plant tissues^1–7^, potentially acting as signals^8–12^. Transport of mRNAs between cells and tissues has been shown to play a role in several physiological and developmental processes in plants, such as tuberization^13^, leaf development^14^ and meristem maintenance^15^; yet for most mobile mRNAs, the biological relevance of transport remains to be determined^16–19^. Here we perform a meta-analysis of existing mobile mRNA datasets and examine the associated bioinformatic pipelines. Taking technological noise, biological variation, potential contamination and incomplete genome assemblies into account, we find that a high percentage of currently annotated graft-mobile transcripts are left without statistical support from available RNA-seq data. This meta-analysis challenges the findings of previous studies and current views on mRNA communication.

## Abstract (web summary)

This study reveals that a substantial number of transcripts that are currently annotated as graft-mobile lack statistical support from available RNA-seq data.

## Main

**Fig. 1:**
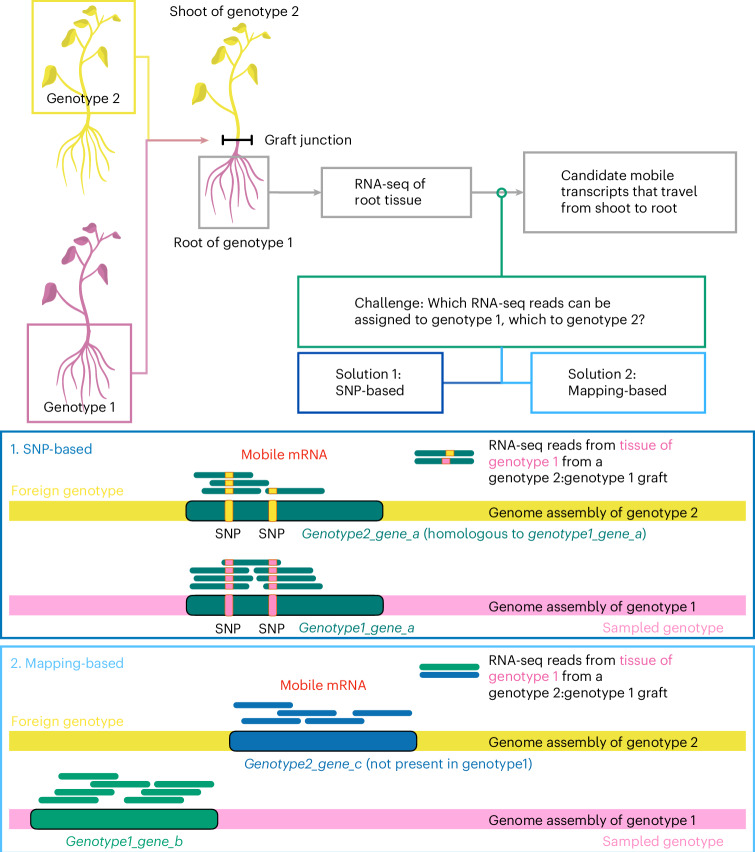
Grafting coupled with RNA-seq to identify transcripts that move from tissue of one genotype/species/ecotype/cultivar into tissue of another across the graft junction. Grafting-based strategy for identification of mRNAs that move from shoot (scion) to root (stock), from genotype 2 to genotype 1, using a scion:stock = genotype 2:genotype 1 heterograft. The same strategy can be used to identify transcripts that move from shoot to root from genotype 1 to genotype 2 using a genotype 1:genotype 2 graft. Transcripts that move root to shoot can be identified by analysing mRNAs in shoot tissue. Natural grafts, such as those established between the parasitic dodder plant and its host plants, can be used in place of artificial grafts. A key challenge in all such approaches is how to assign transcripts to each genotype; methods for doing so are based on (**1**) SNP identification or on (**2**) the alignment to different reference genomes. For grafts from the same species, or similar genotypes, SNPs can be used to distinguish between genotypes and thus identify the source genotype of each transcript (**1**). For grafts between different species, mapping (**2**) each RNA-seq read to the genome assemblies can be an effective method for determining which transcripts are specific to one species.

A key step in mobile mRNA studies is the assignment of RNA-seq reads to different genotypes. One way of identifying the genotype is based on single nucleotide polymorphisms (SNPs) (Fig. 1). Typically, a requirement is made for a defined number of RNA-seq reads to have a SNP that corresponds to the alternative allele for a transcript to be assigned to a foreign genotype. Published criteria are: ≥1 RNA-seq read covering at least two SNPs^3^, ≥2 reads^3^, ≥3 reads^20^ or >3 reads^2^ covering a single SNP. When these criteria are met, the corresponding transcript is defined as mobile.

**Extended Data Fig. 1:**
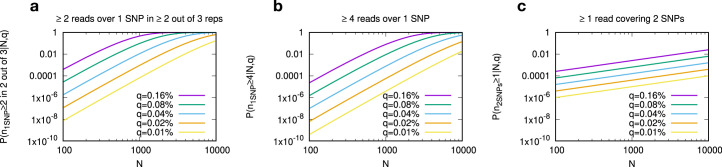
Published criteria for defining mobile mRNAs based on absolute read counts suffer from read-depth dependencies. These plots show the probabilities of transcripts being defined as mobile by chance. Three different mobile mRNA definitions (**a, b, c**) and their dependence on read-depth (N) and on the rate of a SNP matching to the alternate allele (q) are depicted. The number of read counts over one SNP that correspond to the alternate allele is denoted by n1SNP, over two SNPs by n2SNPs. The probabilities were calculated using a cumulative binomial distribution, that is we account only for the nucleotides that correspond to the two alleles of interest. Note that both axes are on a log-scale. The requirement for co-occurring SNPs on one read (**c**) is more stringent and less likely to occur by chance at higher read-depths. For low values of q, these criteria are robust up to moderately high (several hundred) read-depths and would be unlikely to occur by chance.

As previously reported^21^, criteria based on absolute numbers of reads, such as those above, exhibit a read-depth dependency (Extended Data Fig. 1). This is a consequence of sequencing noise.

Illumina sequencing machines produce base-calling errors at a rate of ~0.1–1% per base^22,23^. Sequencing providers often provide a quality assurance, for instance, that 85% of the reads have a Phred quality score of at least Q30 (that is, a base-calling error of less than 10^−3^ = 0.1%). However, base-calling inaccuracies are not the only source of error. Before sequencing, reverse transcriptases can introduce base changes with an error rate of ~0.001–0.01%; the reverse transcription reaction error may exhibit a nucleotide bias, for instance, ‘G’ to ‘A’^24,25^, and a range of other artefacts^26^. On average, 6.4 ± 1.24% of sequences are mutated^22^. The average error rate of next-generation sequencing technologies has been estimated as 0.24 ± 0.06% per base^22,27^, with RNA-seq errors tending to be higher^27^.

**Fig. 2:**
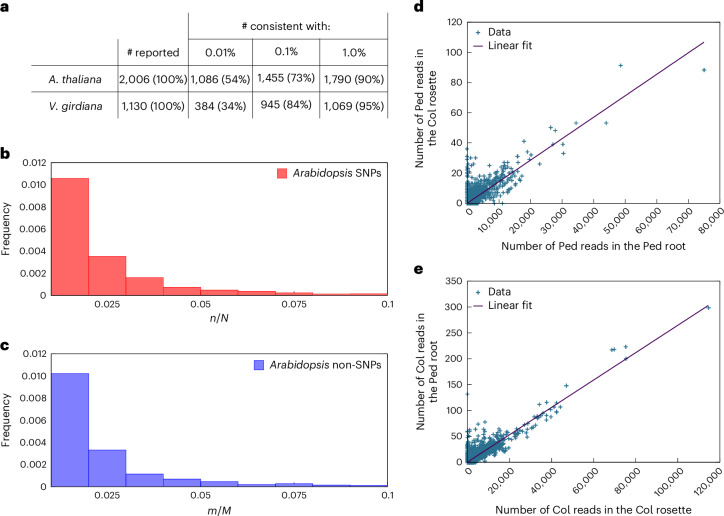
Alternative interpretations for the evidence for mobile mRNAs. **a**, Total numbers of reported mobile mRNAs in *Arabidopsis thaliana*^2^ and *Vitis girdiana*^3^ that can be explained by expected sequencing noise. Two values for the probability of the sequenced nucleotide at a SNP position being assigned to an alternative allele are given: 0.01% and 0.1%. **b**,**c**, The distributions of nucleotides at SNP and other positions (‘non-SNP’) can be informative for evaluating the evidence for the alternative allele. **b**, Histograms of the ratio of the number reads that match the alternative allele, *n*, over the number of reads of local and foreign reads, *N*, for each SNP position in the mobile population on examples from *Arabidopsis*^2^. Several SNPs have reads that match the alternative allele (*n*/*N* > 0). **c**, Histograms of the ratio of the number reads that match the second most frequent nucleotide, *m*, over the sum of the number of reads over the most frequent and second most frequent nucleotide, *M*, for neighbouring positions to SNPs. An exact two-sample Kolmogorov–Smirnov test does not find significant differences in the distributions over SNPs and other positions (*D* = 0.089302, *P* = 0.3575). A Welch two-sample *t*-test (*P* = 0.6421), Wilcox rank sum test (*P* = 0.6388) or an exact two-sample Kolmogorov–Smirnov test (*P* = 0.4065) does not support the values for SNPs being higher than other positions. **d**,**e**, Of the 2,006 previously identified mobile mRNAs^2^, 953 unique mobile mRNAs were found in only 1 replicate in different organs of an adult Ped-0:Col-0 (root:shoot) graft. Such high numbers are not consistent with our hypothesis of sequencing noise and biological variation. Investigating the reciprocal relationship between root alleles that were detected in the rosette (**d**) and vice versa (**e**), in the root (1,373 mRNAs/867 unique) and rosette (577 mRNAs/151 unique) samples, identifies a strong linear correlation (*P* = 2 × 10^−16^) between expression in the source tissue and potential mobility. Interestingly, those SNPs that lie towards the lower read depth in each plot deviate the most from the linear relationship. However, these transcripts have low read numbers only in the ‘source’ tissue, whereas they have high read numbers in the sampled tissue and reads over SNPs that are consistent with sequencing errors. These plots thus show two effects: sequencing noise + either non-selective transport across the whole transcriptome or contamination.

We therefore investigated whether noise in RNA-seq may influence the identification of mobile mRNAs. Figure 2a lists how many reported mobile mRNAs have numbers of reads with SNP occurrences that are consistent with an assumed error rate^21,28^. As an example, for an accuracy of SNP calling of 99.97% (that is, 0.03% sequencing noise, Phred score Q35, and an error probability for the alternative allele of ~0.01%), the evidence for 1,086 out of 2,006 (54%) and 384 out of 1,130 (34%) previously identified mobile mRNAs^2,3^ is in line with what would be expected from sequencing noise (Fig. 2a).

One way to increase the accuracy of detecting foreign transcripts is to consider multiple SNPs per read. If SNPs are located closely together, then a single RNA-seq read may cover more than one SNP. Accounting for co-occurring SNPs on the same read leads to the multiplication of their probabilities, resulting in higher accuracy (less likely to occur by chance), less pronounced read-depth dependence than single SNP criteria (Extended Data Fig. 1) and greater confidence in these reads being from a foreign genotype. We therefore examined reads over co-occurring SNPs (Extended Data Fig. 3 and Supplementary Table 1). In the *Arabidopsis* homograft datasets^2^, we found a total of 1,753,179 reads covering more than 1 SNP in the root and 1,977,539 in the shoot of Col-0; of these 1,675 (0.10%) and 1,797 (0.091%), respectively, had reads supporting the alternative allele for at least 1 but not all SNPs. These inconsistent calls are in line with the notion that sequencing noise may confound the identification of mobile mRNAs. We found 29 reads (1.6 × 10^−3^%) in the root and 2 reads (1.0 × 10^−4^%) in the shoot for which all SNPs supported the alternative allele. Interestingly in Ped-0 homograft data, the proportion of reads with full support for the alternative allele was significantly higher (0.038% in the root, 0.12% in the shoot). Investigating these co-occurring SNPs revealed another confounding factor in the identification of mobile mRNA; several loci showed apparent heterozygosity in the Ped-0 ecotype (Extended Data Fig. 4).

**Extended Data Fig. 2:**
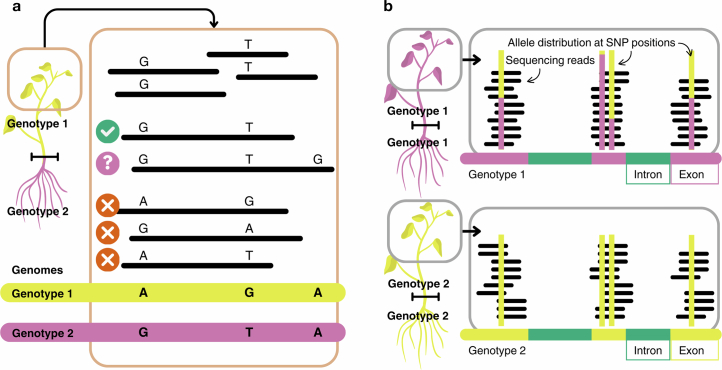
Allelic differences in multiple SNPs per read and the appearance of heterozygosity (in homozygous species) can be used to check the viability of SNPs and exclude potentially problematic transcripts from the analysis. **a**, SNPs can be in close proximity, and therefore it can happen that several SNPs are recorded in the same RNA-Seq read. In this example, genotype 1 has three SNPs very close to each other: A, G and A (yellow bar). In genotype 2, we find G, T, A (magenta bar) in those positions. In this schematic example, reads from the shoot of Genotype 1 are mapped to Genotype 2. If all covered loci carry the allele of Genotype 2, we are observing evidence for the read being from Genotype 2 and the associated transcript being potentially mobile. On the other hand, if only one loci carries the allele of Genotype 2, the outcome is inconclusive, as it may indicate sequencing errors. **b**, A. thaliana is a selfing species, so we expect homozygocity at all positions for all reads mapping to the genome at all positions. However, for duplicated genes (magenta) in Genotype 1, which may be single copy genes in Genotype 2 (yellow), short read sequencing and mapping to Genotype 2, can give rise to what appears to be heterozygocity. When there are two alleles present in the homograft data (magenta and yellow), we may be observing pseudo-heterozygocity. See also Extended Data Figure 3.

**Extended Data Fig. 3:**
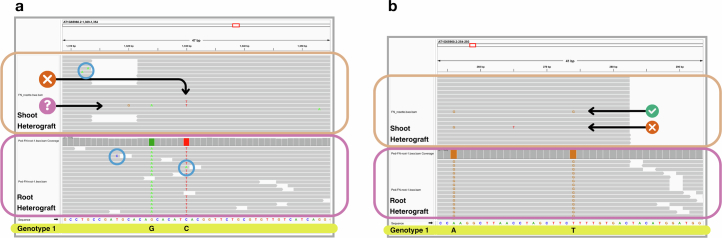
Examples of co-occurring SNPs. **a**, There are two SNPs, G and C in Genotype 1 (Col) and A and T in Genotype 2 (Ped). These are likely two sequencing errors in the root sample, a C at a non-SNP position and an A at a SNP position (both highlighted in blue circles). In the shoot sample we see potential evidence for mobility at the SNP level but in one case the second SNP is not present and in the other case another sequencing error has occurred (G). Three further sequencing errors (two As on the top left, one A on the right) are also present in the shoot. **b**, This example shows two positions, A and T in Genotype 1 (Col) and G and G in Genotype 2 (Ped), for which some reads support the alternate allele (green tick), whereas others are likely sequencing errors (red cross). In the latter case, one G is in the correct position but the other G is not present and a further mismatch (T) has occurred. See Figure 2 for further explanations. The images are annotated screenshots taken in IGV^37^. Data taken from^2^.

**Extended Data Fig. 4:**
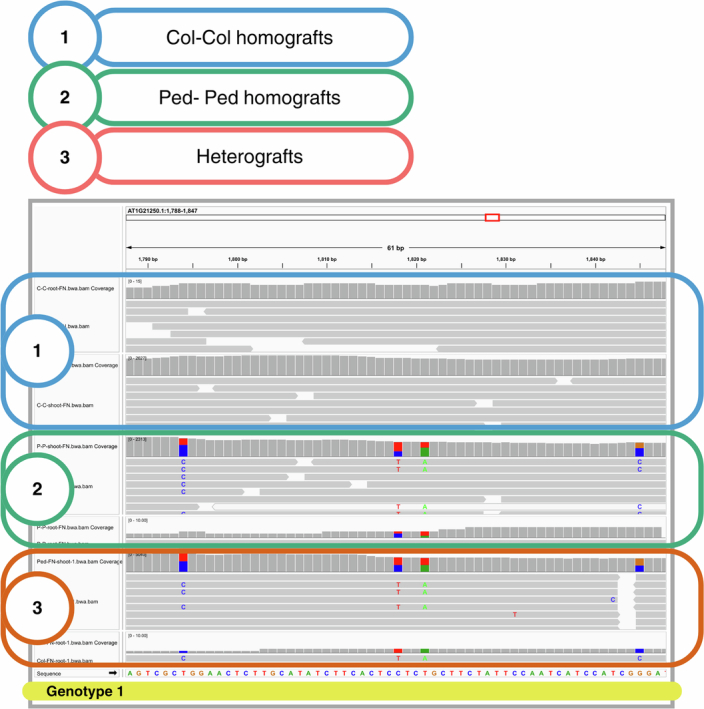
A number of genes in Ped-0 show apparent heterozygosity, both in the homograft (2) and heterograft (3) datasets. At the highlighted positions there are distinct populations of alleles (depicted as red/blue and red/green bars). This is possibly due to the gene being duplicated in Ped, resulting in pseudoheterozygocity.

Such apparent heterozygosity could be caused by a lack of introgression or gene copy-number variation; it has been estimated that 10% of the annotated genes in *Arabidopsis* have copy-number variation^29,30^. Differences in gene copy numbers can lead to reads not mapping correctly, which gives rise to pseudo-SNPs and pseudo-heterozygosity^29,30^. Of the 2,570 genes assigned as pseudo-heterozygous^30^, we found 188 mobile transcripts^2^ (Extended Data Figs. 2 and 4). We identified 19 transcripts in the Ped-0 samples that are likely caused by mismapping; interestingly, these include transcripts that frequently fulfill the criteria for being classified as mobile (Supplementary Table 3). Thus, in addition to technological noise, there are also biological causes that could be falsely interpreted as SNPs of an alternative allele. As a consequence, it becomes important to not rely solely on Phred scores for estimating errors in SNP assignments. We next sought to estimate this background noise level, that is, the frequency for finding the alternative allele when the alternative allele is not actually present. This value can be estimated from available *Arabidopsis* homograft data^2^. We counted the number of RNA-seq reads in the homograft with a SNP that matched the foreign genotype. For *Arabidopsis* homograft datasets (ecotypes Col-0 and Ped-0), these background noise levels were 0.084% (Col-0:Col-0 root), 0.082% (Col-0:Col-0 shoot), 0.68% (Ped-0:Ped-0 root) and 0.51% (Ped-0:Ped-0 shoot). The higher background error rate in Ped-0 is consistent with more Col-0 transcripts being identified as mobile in sampled Ped-0 tissue^2^. For an average background error rate of 0.34%, we find that over 1,455 out of 2,006 (>73%) and over 945 out of 1,130 (>84%) of annotated mobile mRNAs would not be distinguishable from expected errors (Fig. 2a). Consistent with this, poor overlap between experiments has been noted^18,31^, orthologues in closely related species exhibit conflicting mobility, and reported low ratios of mobile to endogenous mRNAs^3,5,7^ are in line with the level of noise.

Another way to distinguish noise from potential evidence for the alternative allele is to investigate the differences in nucleotide distributions at SNP positions compared to other positions in the sequence (non-SNP positions). If a second genotype were present, we would expect the distribution of nucleotides at any SNP position to be enriched in the nucleotide that supports the alternative allele. Furthermore, mRNAs that are transported to cells with low endogenous level (potential signals) would have a value of *n*/*N* close to 1, where *n* is the number of reads that match the alternative allele and *N* the total number of reads (endogenous + foreign). We investigated the distribution of *n*/*N* for each SNP in the mobile population of *Arabidopsis*^2^. While we do not find evidence for *n*/*N* values close to 1, there are non-zero values of *n*/*N* that seem to support the presence of the alternative allele (Fig. 2b). However, looking at all neighbouring positions of SNPs and computing the number of reads with the second most frequent nucleotide, *m*, over the sum of the most frequent and second most frequent nucleotides, *M*, we find no support for the SNP positions being different (*P* = 0.3575) (Fig. 2c). Thus, the expected shift in the distribution towards higher *n*/*N* values, that is *n*/*N* > *m*/*M*, is not observed. Given the low prevalence, it is important to note that this analysis does not exclude there being instances, potentially even thousands, of reads with SNPs associated with mobile mRNAs in the data, but if so we cannot distinguish them from noise.

Interestingly, two samples from *Arabidopsis*^2^ do contain numbers of foreign reads that exceed expected noise levels. Investigating further, we find that these samples exhibit a strong linear correlation between the read counts of the grafted tissues (Fig. 2d,e). Similarly, *Arabidopsis* transcripts found in *Cuscuta pentagona* correlate with the expression levels in the host genotype^1^. Finding constant proportions of a whole transcriptome is indicative of contamination. Another explanation is that the whole transcriptome is transported, with detection being proportional to read depth. Given the available data, we cannot distinguish between these possibilities.

**Extended Data Fig. 5:**
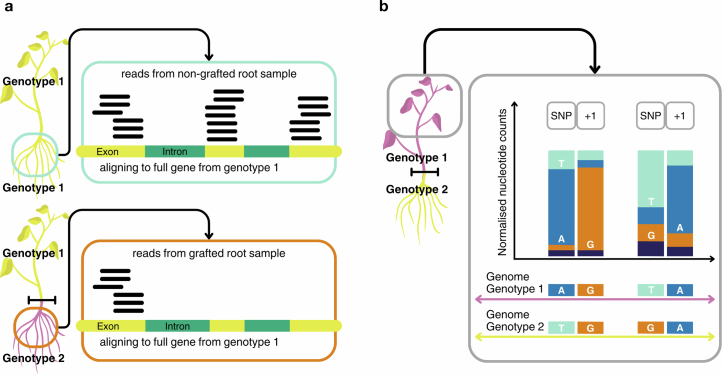
Full-length transcript coverage and differences in the distribution of nucleotides between SNPs and other positions enhance the evidence for the presence of a foreign transcript in the sampled tissue. **a**, Sequenced transcripts would ideally have RNA-Seq reads covering most of the sequence, that is that all exons of the mRNA are approximately equally covered by sequenc- ing reads (top left). Reads covering all exons in the sample from Genotype 2 provide support for the whole transcript having moved from Genotype 1 to Genotype 2 across the graft junction. Transcripts with coverage only for a subsequence (bottom left) do not support full-length presence of the for- eign transcript. **b**, Neighbouring positions to SNPs can be used as a negative control to evaluate the strength of the signal at SNP positions. Shown here are neighbouring positions of the identified SNPs at the next nucleotide (SNP position +1). If the neighbouring position shows similar levels of alter- native nucleotides as the SNP position, the these are likely sequencing errors, rather than evidence for the alternate allele. If the SNP positions have a different frequency of Genotype 2 allele than the neighbouring position has errors, then there is evidence for the alternate allele. Analysing the fre- quencies of nucleotides at known SNP positions and their neighbours can aid data interpretation.

**Extended Data Fig. 6:**
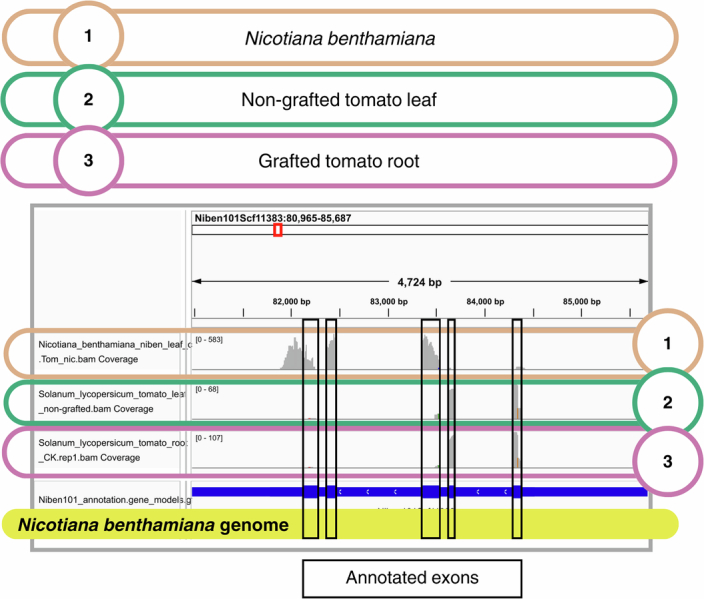
An example of poor coverage for a candidate mobile mRNA. In the Nico- tiana benthamiana annotation of the depicted gene (Niben101Scf11383g00015.1) we find 5 annotated exons of which all are populated with reads at different levels (grey histograms). In the samples from tomato, non-grafted or grafted we see that not all annotated exons are populated with reads and that the exons with coverage are populated in both grafted and non-grafted samples. Coverage over the full length of the mRNA may help reduce the risk of reads mapping to isolated regions being potentially misinterpreted, Extended Data Figure 5. This is a screenshot taken in IGV^37^.

Approaches that do not rely on SNPs, such as for cross-species studies, might avoid some of the above issues. A typical pipeline for analysing between-species grafts first maps reads to the reference genome of the sampled tissue (genotype 1 in Fig. 1). Unmapped reads are then compared to the reference genome of the potential source tissue (genotype 2 in Fig. 1). The success of this approach depends on the quality of the genome assembly. Supplementary Table 2 lists some genome completeness estimates for assemblies that were used in previous mobile mRNA studies. For instance, at the time of the study that investigated the movement of transcripts from a *Nicotiana benthamiana* scion to a *Solanum lycopersicum* (tomato) rootstock^6^, ~15% of the genome was not yet assembled (Extended Data Fig. 6). The authors therefore collected RNA-seq data and applied stringent mapping criteria to mitigate effects of using an incomplete assembly. However, repeating their procedure, we found that many reads that did not map to the tomato genome all aligned to small regions of the *N. benthamiana* genome, and that coverage was highly uneven over exons (Extended Data Figs. 5 and 6). Furthermore, blasting the reads identified as being from *N. benthamiana* against the whole NCBI nucleotide database resulted in 100% matches to highly conserved sequences contained within many genomes, including *N. benthamiana* and other Solanaceae species, in particular to 18S ribosomal RNA genes, which accounted for 97.7% of the blast hits to *N. benthamiana* (Extended Data Fig. 8). To test for false negatives, we mapped the heterograft reads directly to the *N. benthamiana* genome and found 16 short transcripts that could not be distinguished between genomes (Supplementary Table 4).

**Extended Data Fig. 7:**
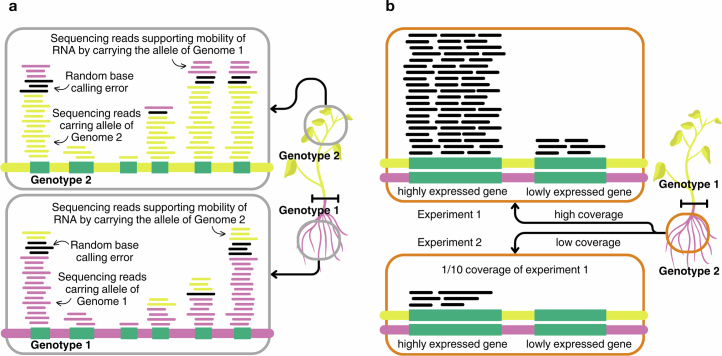
Challenges in identifying non-selective mobility versus contamination in high-throughput mobile mRNA detection using RNA-seq data in within-species grafts and cross-species grafts. (**a**) The presence of Genotype 1 reads in Genotype 2 samples and vice versa, across the whole of genome, especially in genes expressed in both tissues is consistent both with non- selective transport and contamination. (**b**) The two genes presented in this schematic figure have different relative expression levels. In Experiment 2 the sequencing depth is insufficient to detect lowly expressed genes.

**Extended Data Fig. 8:**
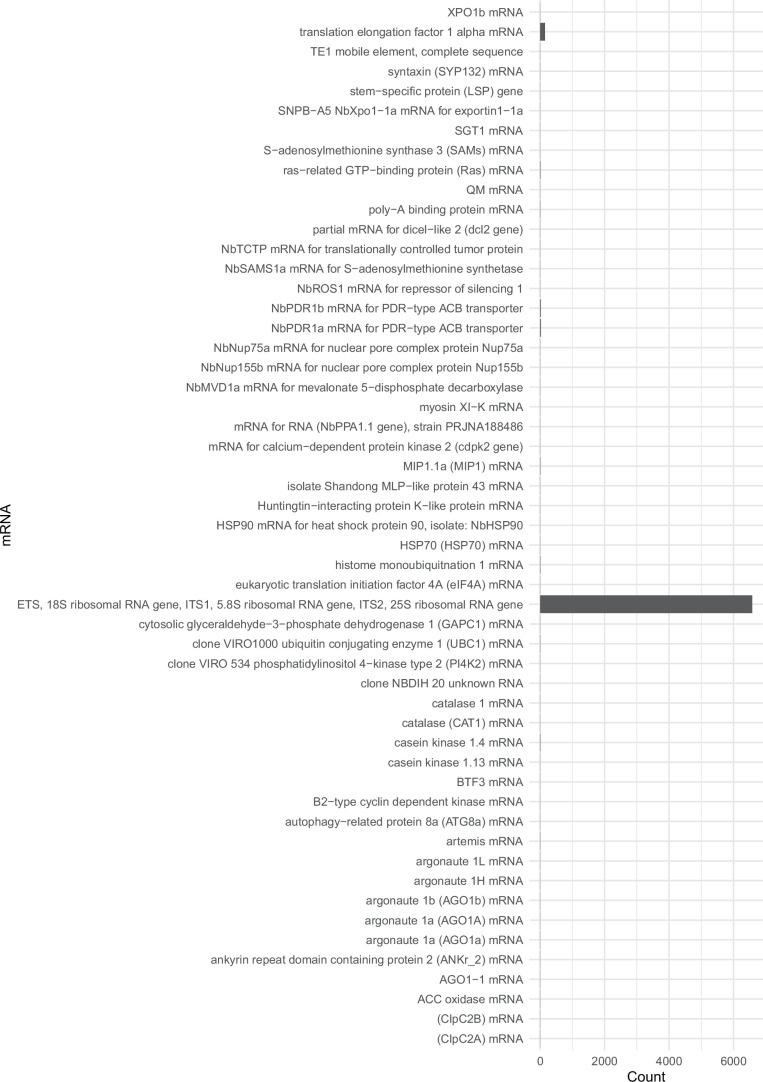
**A bar plot of the blast results of unmapped reads against the NCBI database that matched Nicotiana benthamiana.**

In addition to genome assembly quality, read depth can also bias the interpretation of RNA-seq data from grafts between different species (Extended Data Fig. 7). For instance, ~30% of the *Arabidopsis thaliana* transcriptome was reported to move into *Cuscuta pentagona*, while only 9% of the tomato transcriptome moves to *Cuscuta*^1^. However, there is a large discrepancy in the amount of RNA-seq data between tomato (6 Mb) and *Arabidopsis* experiments (2 Gb). Greater coverage would be expected to lead to more transcripts being detected^32–34^, thus explaining the reported bias in mobility between species.

Overall, our study raises questions about published numbers of mobile mRNAs. The experimental evidence for movement of a small number of mRNAs over long distances in plants is compelling^5,6,11,15,17,35,36^. However, on the basis of RNA-seq studies, several thousand mobile transcripts have been reported^1–4,6,7^. Here we question this extrapolation from tens of validated cases to the published vast numbers of potential long-distance signalling agents.

## Recommendations

**Fig. 3:**
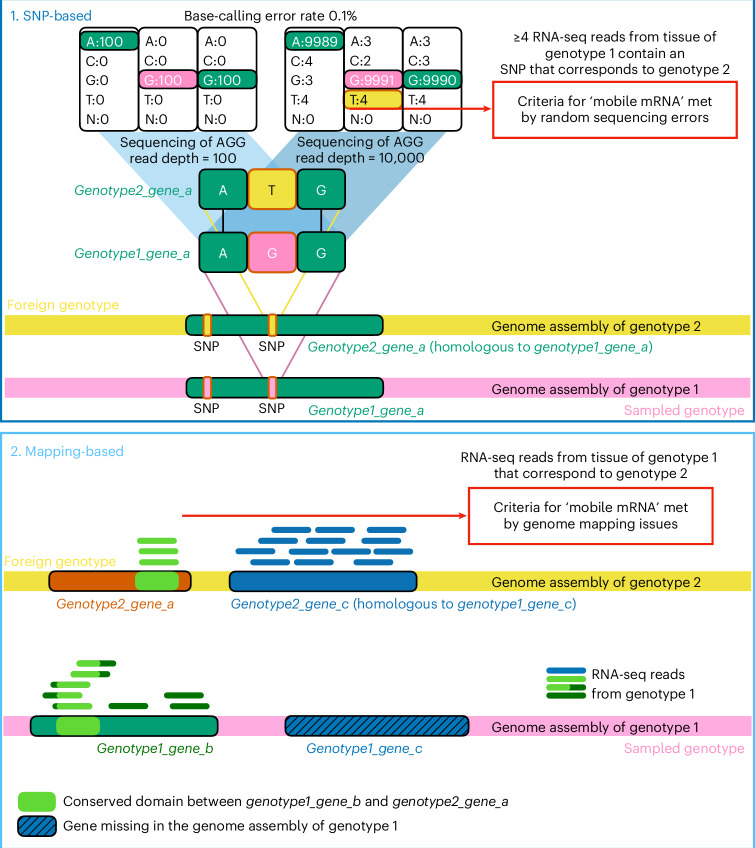
Mobile mRNA identification is not without challenges. (**1**) Technological noise can lead to challenges in the assignment of RNA-seq reads. In SNP-based methods of mobile mRNA detection, it is important to be able to differentiate between sequencing-associated errors and genuine SNPs. In the above case, an RNA-seq read with a ‘T’ at the SNP position would be indicative of the read having come from the alternative allele, genotype 2. However, every position has an error rate and the higher the read depth, the more incorrect base calls are to be expected. Base changes could arise for reverse transcriptase or amplification steps, although their error rate is typically orders of magnitude lower than sequencing errors. Conserved regions in gene families can give rise to similar challenges in distinguishing mapping ambiguities from genuine SNPs. Defining an mRNA as being mobile based on thresholds of RNA-seq reads that contain a SNP can result in base-calling errors and mapping ambiguities biasing the interpretation. To reduce the risk of such events occurring, further stringent filters can be applied (for instance, using only SNPs that are bi-allelic^2^) or applying rigorous statistical comparisons (for instance, estimating the allele calling frequencies and comparing them between homograft and heterograft^21^). (**2**) Genome complexity and genome quality can lead to mapping challenges. Orthologous sequences (light green) can result in some RNA-seq reads aligning to a different gene and different genotype. Genome assemblies that are not complete (telomere to telomere) from exactly the same genotype as used for grafting can result in potential mismappings. The shaded blue gene in genotype 1 is missing in the reference genome assembly, resulting in RNA-seq reads from this transcript being mapped to genotype 2.

We described several challenges in identifying mobile mRNAs from short-read RNA-seq data (Fig. 3). While we do not present solutions, we suggest checks that can be performed to reduce the risk of false positives. We thus end with a list of recommendations. We assume that experimental issues have been taken care of, such as checking the samples for cross-contamination, verifying that graft junctions form functional vascular connections, and every effort has been made to use high-quality genome assemblies.

1. SNP reliability. A genome mapping visualization tool such as IGV^37^ can be used to check for pseudo-heterozygosity and contamination in the samples. Observing the distribution of nucleotides at potential SNP positions and comparing to other positions can provide confidence in the SNPs and the alternative allele calls. These distributions should be compared to those from homograft data.
2. Co-occurring SNPs. RNA-seq reads that cover multiple SNPs can be used to check whether the SNPs that are associated with a certain genotype co-occur in such reads. Long-read and direct RNA sequencing have higher error rates but would allow the full transcript with all SNPs to be assessed. Sequencing protocols that barcode individual molecules by using adapters with unique molecular identifiers (UMIs) can be used to determine the error rates and check whether all reads from the same molecule are consistent in terms of their genotype assignment.
3. Accuracy of experimental and computational procedures for identifying foreign RNA-seq reads. Calculating the ratio of the number of RNA-seq reads assigned to an alternative allele (foreign reads) over the total number of mapped RNA-seq reads for an experiment (foreign + endogenous reads) is a useful metric. This value should be computed for homografts and compared to the value calculated from heterograft data.
4. Reproducibility and consistency of putative mobile transcripts. Independent biological replicates should be used to characterize the inherent variability in the identification of candidate mobile transcripts. Reciprocal grafting is recommended to evaluate whether mobile mRNA and their orthologues are consistently mobile (if mobility motifs are inherent to transcripts, then near-identical sequences would be expected to also be mobile) and, if not, potentially pinpoint determinants of mobility.
5. Alternative hypotheses. Definitions for mobile mRNAs using non-validated criteria are best avoided. It is important to test different hypotheses (for example, SNP vs sequencing noise; read from a foreign genotype vs mapping error; transport vs contamination; signalling molecules vs leftovers from differentiating cells) to explain the data. The plausibility of associated mechanisms can lend weight to different hypotheses.

## Methods

All code and scripts are freely available from our GitHub repository at https://github.com/mtomtom/reanalysis-mobile-mrna/tree/main (ref. ^38^).

### RNA-seq data processing

The raw reads were mapped to the references using hisat2 (v.2.1.0)^39^,


hisat2 -x genome -1 read1 -2 read2 > mapping.sam


and processed using samtools (v1.9)^40^,

samtools sort -o mapping.bam mapping.sam

samtools index mapping.bam

### Expression level quantification

The expression levels were quantified with Stringtie (v.1.3.5)^41^ using


stringtie mapping.bam -e -G genes.gff -o output.gtf -A output.abundance.txt


### Quantification of raw counts of all nucleotides

The raw counts were quantified with bcftools (v.1.10.2)^40^ using

bcftools mpileup -A -q 0 -Q 0 -B -d 500000

--annotate FORMAT/AD, FORMAT/ADF, FORMAT/ADR, FORMAT/DP, FORMAT/SP, INFO/AD,

INFO/ADF, INFO/ADR

These flags were chosen to compare the raw error rates between the homograft and heterograft to catch all nucleotides. Note that the bcftools mpileup default sequencing depth is 8,000, but the most highly expressed genes have up to 200,000 reads covering a locus within the datasets we considered.

### Blast search

The NCBI nucleotide database was downloaded on 21 October 2022 and blast+ (v.2.9.0)^42^ was utilized for alignments using

blastn -db nt -query unmapped.fasta -max_target_seqs 10 -max_hsps 1-evalue 1e-25

-outfmt '6 qseqid sseqid pident evalue staxids sscinames scomnames sskingdoms stitle'

### Estimating the accuracy of mobile mRNA detection

If we are only interested in the number of reads that contain a SNP that corresponds to the alternative allele, we can use a binomial distribution (*q* is the probability the SNP matches the alternative allele, 1 − *q* is the probability that it does not) to evaluate the probability of this event occurring by chance^21^. The probabilities of errors occurring by chance were calculated from a standard cumulative binomial distribution, *P*(*k* ≥ *m*|*N*) = 1 − *P*(*k* < *m*∣*N*), which accounts for the requirement of having *k* reads, where *k* is at least *m*, out of *N*. Considering replicates can be handled in the same way (the probability of each SNP is computed from the cumulative binomial function and the requirement for a defined number of replicates can likewise be computed from a cumulative binomial function). Multiple SNPs per read results in a multiplication of probabilities. Cumulative binomial function values were computed using standard available functions in Python and R.

### Assessing how many SNPs can be explained by sequencing-associated errors

Rather than ‘defining’ a transcript as mobile, we evaluated the probability of the data being consistent with expected noise against the probability of the data being best explained by the presence of two genotypes (and therefore potential candidates for mobile transcripts)^21^. Essentially, this means that if we find 10 out of 100 reads that match the alternative allele, we compute how likely this would occur by chance for a defined error rate. The implicit but rarely checked assumption in all SNP-based mobile mRNA detection pipelines is that the occurrence of reads that support the alternative allele in the heterograft data is larger than in homograft data. The uncertainty in the inferred error rate depends on the amount of data. We capture this uncertainty through probability distributions to inform inferences drawn from the data^21^. This ratio of the statistical evidence of one hypothesis over another is known as the Bayes factor^43^. The classifications in Fig. 2a are based on the commonly used value of log Bayes factor greater than 1 (refs. ^21,43^). The statistical comparison of error rates was performed using baymobil^28^.

### Statistics for comparing nucleotide distribution as SNP positions vs other positions

To compare the full distributions of *n*/*N* and *m*/*M* values for different positions of RNA-seq reads, we used an exact two-sample Kolmogorov–Smirnov test, ks.test, available in R^44^. To evaluate whether the data supported the SNP distributions having higher values of *n*/*N* than other positions (*m*/*M*), we used an asymptotic two-sample Kolmogorov–Smirnov test. These tests were carried out for histograms with 100 bins.

### Pseudo-heterozygosity

We downloaded the pseudo-heterozygous data from https://zenodo.org/records/6025134 (ref. ^30^). From the vcf-file we extracted all heterozygous calls for accession 9947 (Ped-0) and obtained 6,303 heterozygous SNPs. We compared these SNPs against the MATRIX_GWAS_raw_position.txt (from 10.5281/zenodo.5702395). We intersected these potential duplicate genes with the list of mobile genes^2^ and found 19 duplicate genes. These are given in Supplementary Table 3.

### Genome assembly completeness estimation

We downloaded all the assemblies mentioned in the original papers and estimated their completeness with Abyss (v.1.9.0) using the command ‘abyss-fac’.

### Contamination analysis

We analysed the samples of the root (1,373 mRNAs/867 unique) and rosette (577 mRNAs/151 unique), and reciprocally inspected the relationship between root alleles that were detected in the rosette and vice versa. We took the raw sequencing depth for 48,934 previously identified SNPs. For each SNP, we plotted the number of reads with a rosette allele (Col-0) found in the root sample (Ped-0) against the number of reads with the same SNP in the rosette sample. Similarly, we plotted the number of reads with the root allele (Ped-0) found in the rosette sample (Col) against the number of reads with the endogenous SNP (Ped-0) in the root sample. The linear fit was performed within gnuplot^45^.

### Reporting summary

Further information on research design is available in the Nature Portfolio Reporting Summary linked to this article.

## Supplementary information

Reporting Summary

Supplementary Table 1 RNA-seq reads containg multiple SNPs.

Supplementary Table 2 Completeness of genome assemblies used in mobile mRNA studies.

Supplementary Table 3 Potentially pseudo-heterozygous genes in the *Arabidopsis* Ped-0 ecotype and their functional annotations.

Supplementary Table 4 Potentially false negatives in the heterograft between *Solanum lycopersicum and Nicotiana benthamiana*.

## Data Availability

We used the following published datasets and the archived reads from NCBI: *Cuscuta pentagona*^1^ (PRJNA257158; this dataset was incomplete and partly corrupt); *Vitis vinifera*^3^ (SRP058158 and SRP058157); *Solanum lycopersicum, Nicotiana benthamiana*^6^ (SRP111187); *Arabidopsis thaliana*^2^ (PRJNA271927). We used deposited supplementary datasets of the associated publications to obtain the numbers of identified mRNAs. For each of the graft studies, we downloaded the reference genome sequence that matched the one that was used in the original paper with the same annotations; most are publicly available in Ensembl plants^46^.

## Code availability

We used largely available software packages as stated in the Methods. All code and scripts are freely available on GitHub at https://github.com/mtomtom/reanalysis-mobile-mrna/tree/main (ref. ^38^).
