# Supplementary material for: Re-analysis of mobile mRNA datasets raises questions about the extent of long-distance mRNA communication
Source: Nat Plants. 2025 Apr 16;11(5):977–84. doi: 10.1038/s41477-025-01979-x (PMC12095074; doi:10.1038/s41477-025-01979-x)
Supplement: Supplementary file 2 — RNA-seq reads containg multiple SNPs. [file 41477_2025_1979_MOESM2_ESM.pdf]

Supplemental Table. S 1 RNA-Seq reads containg multiple SNPs. The numbers of reads covering more than 1 SNP (2nd column) in the Arabdopsis dataset [2]. There are instances where some but not all SNPs support the alternate allele (3rd column, mixed SNPs and inconsistent genotype calls), and instances for which all SNPs support the alternate allele (4th column, consistent foreign genotype call). All numbers based on raw data, prior to SNP quality filtering that is often applied in various pipelines.

Dataset	Reads with > 1 SNP	Mixed SNPs	All foreign SNPs
CCRootFN	1753179	1675	29
CCShootFN	1977539	1797	2
ColFNRoot1	832072	674	81
ColFNRoot2	834696	677	45
ColFNRoot3	891477	755	43
ColFNShoot1	838014	721	287
ColFNShoot2	1366447	1243	108
ColFNShoot3	812450	651	429
PPRootFN	610762	759	235
PPShootFN	366704	458	455
PedFNRoot1	557997	598	863
PedFNRoot2	467877	538	1996
PedFNRoot3	477536	589	1244
PedFNShoot1	339750	564	240
PedFNShoot2	496332	735	1730
PedFNShoot3	444793	631	520
