# Supplementary material for: Re-analysis of mobile mRNA datasets raises questions about the extent of long-distance mRNA communication
Source: Nat Plants. 2025 Apr 16;11(5):977–84. doi: 10.1038/s41477-025-01979-x (PMC12095074; doi:10.1038/s41477-025-01979-x)
Supplement: Supplementary file 3 — Completeness of genome assemblies used in mobile mRNA studies. [file 41477_2025_1979_MOESM3_ESM.pdf]

Supplemental Table. S 2 Completeness of genome assemblies used in mobile mRNA studies. These assemblies are constantly being improved and newer versions have been published. It is the completeness in gene-rich regions that is important for aligning transcripts, not the overall completeness.

	Genome size	Assembly size	%Assembled	Year of Publication
A. thaliana Col-0, TAIR10 [48]	135 Mb	119.5 Mb	88.5%	2012
N. benthamiana [49]	3 Gb	2.412 Gb	80.4%	2012
S. lycopersicum [50]	900 Mb	760 Mb	84.4%	2012
V. vinifera [51]	500 Mb	473.8 Mb	94.8%	2007
