# Supplementary material for: Re-analysis of mobile mRNA datasets raises questions about the extent of long-distance mRNA communication
Source: Nat Plants. 2025 Apr 16;11(5):977–84. doi: 10.1038/s41477-025-01979-x (PMC12095074; doi:10.1038/s41477-025-01979-x)
Supplement: Supplementary file 4 — Potentially pseudo-heterozygous genes in the Arabidopsis Ped-0 ecotype and their functional annotations. [file 41477_2025_1979_MOESM4_ESM.pdf]

Supplemental Table. S 3 Potentially pseudo-heterozygous genes in the Arabidopsis Ped-0 ecotype and their functional annotations.

Gene ID	Functional annotation
AT1G07780	PAI1;PHOSPHORIBOSYLANTHRANILATE ISOMERASE 1; TRANSIENT RECEPTOR POTENTIAL 6;TRP6
AT1G21250	ATWAK1;CELL WALL-ASSOCIATED KINASE 1;PRO25;WAK1
AT1G31885	NIP3;1;NOD26-LIKE INTRINSIC PROTEIN 3;1
AT1G56500	SOQ1;SUPPRESSOR OF QUENCHING 1
AT1G61840	Cysteine/Histidine-rich C1 domain family protein;(source:Araport11)
AT1G66880	Protein kinase superfamily protein;(source:Araport11)
AT1G70330	ENT1;ENT1,AT;EQUILIBRATIVE NUCLEOTIDE TRANSPORTER 1
AT3G11880	transmembrane protein, putative (Protein of unknown function DUF2359, transmembrane);(source:Araport11)
AT3G16390	NITRILE SPECIFIER PROTEIN 3;NSP3
AT3G23530	Cyclopropane-fatty-acyl-phospholipid synthase;(source:Araport11)
AT3G27690	DEG13;LHCB2;LHCB2.3;LHCB2.4;LIGHT-HARVESTING CHLOROPHYLL B-BINDING 2; PHOTOSYSTEM II LIGHT HARVESTING COMPLEX GENE 2.3
AT4G31980	PPPDE thiol peptidase family protein;(source:Araport11)
AT5G36230	ARM repeat superfamily protein;(source:Araport11)
AT5G38210	LEAF RUST 10 DISEASE-RESISTANCE LOCUS RECEPTOR- LIKE PROTEIN KINASE-LIKE 3; LRK10L3
AT5G38344	Toll-Interleukin-Resistance (TIR) domain family protein;(source:Araport11)
AT5G41750	Disease resistance protein (TIR-NBS-LRR class) family;(source:Araport11)
AT5G45750	ATRABA1C;RAB GTPASE HOMOLOG A1C;RABA1C
AT5G62810	ATPED2;ATPEX14;PED2;PEROXIN 14;PEROXISOME DEFECTIVE 2;PEX14
AT5G66052	transmembrane protein;(source:Araport11)
