# Supplementary material for: Re-analysis of mobile mRNA datasets raises questions about the extent of long-distance mRNA communication
Source: Nat Plants. 2025 Apr 16;11(5):977–84. doi: 10.1038/s41477-025-01979-x (PMC12095074; doi:10.1038/s41477-025-01979-x)
Supplement: Supplementary file 5 — Potentially false negatives in the heterograft between Solanum lycopersicum and Nicotiana benthamiana. [file 41477_2025_1979_MOESM5_ESM.pdf]

Supplemental Table. S 4 Potentially false negatives in the heterograft between *Solanum lycopersicum* and *Nicotiana benthamiana* [6].

Gene ID
Niben101Ctg13237g00004
Niben101Scf01882g00005
Niben101Scf02124g01035
Niben101Scf02309g03014
Niben101Scf02441g04001
Niben101Scf02485g01006
Niben101Scf04820g03004
Niben101Scf07383t05001
Niben101Scf09223g00005
Niben101Scf10161g00015
Niben101Scf10316g03012
Niben101Scf10324g00002
Niben101Scf10881g00045
Niben101Scf11896g00001
Niben101Scf15618g00001
Niben101Scf17274g00001
